# Withdrawal of inhaled corticosteroids in people with COPD in primary care: a randomised controlled trial

**DOI:** 10.1186/1465-9921-8-93

**Published:** 2007-12-27

**Authors:** Aklak B Choudhury, Carolyn M Dawson, Hazel E Kilvington, Sandra Eldridge, Wai-Yee James, Jadwiga A Wedzicha, Gene S Feder, Chris J Griffiths

**Affiliations:** 1 Centre for Health Sciences, Queen Mary's School of Medicine and Dentistry, Barts and The London, 2 Newark Street, London, E1 2AT, UK; 2 Academic Unit of Respiratory Medicine, Royal Free and University College Medical School, Hampstead Campus, Rowland Hill Street, Hampstead, London, NW3 2PF UK; 3 MRC & Asthma UK Centre in Allergic Mechanisms of Asthma, 5th Floor Thomas Guy House, Guy's Hospital, London, SE1 9RT, UK

## Abstract

**Background:**

Guidelines recommend inhaled corticosteroids (ICS) for patients with severe chronic obstructive pulmonary disease (COPD). Most COPD patients are managed in primary care and receive ICS long-term and irrespective of severity. The effect of withdrawing ICS from COPD patients in primary care is unknown.

**Methods:**

In a pragmatic randomised, double-blind, placebo-controlled trial in 31 practices, 260 COPD patients stopped their usual ICS (median duration of use 8 years) and were allocated to 500 mcg fluticasone propionate twice daily (n = 128), or placebo (n = 132). Follow-up assessments took place at three monthly intervals for a year at the patients' practice. Our primary outcome was COPD exacerbation frequency. Secondary outcomes were time to first COPD exacerbation, reported symptoms, peak expiratory flow rate and reliever inhaler use, and lung function and health related quality of life.

**Results:**

In patients randomised to placebo, COPD exacerbation risk over one year was RR: 1.11 (CI: 0.91–1.36). Patients taking placebo were more likely to return to their usual ICS following exacerbation, placebo: 61/128 (48%); fluticasone: 34/132 (26%), OR: 2.35 (CI: 1.38–4.05). Exacerbation risk whilst taking randomised treatment was significantly raised in the placebo group 1.48 (CI: 1.17–1.86). Patients taking placebo exacerbated earlier (median time to first exacerbation: placebo (days): 44 (CI: 29–59); fluticasone: 63 (CI: 53–74), log rank 3.81, P = 0.05) and reported increased wheeze. In a post-hoc analysis, patients with mild COPD taking placebo had increased exacerbation risk RR: 1.94 (CI: 1.20–3.14).

**Conclusion:**

Withdrawal of long-term ICS in COPD patients in primary care increases risk of exacerbation shortens time to exacerbation and causes symptom deterioration. Patients with mild COPD may be at increased risk of exacerbation after withdrawal.

**Trial Registration:**

ClinicalTrials.gov NCT00440687

## Introduction

Exacerbations in patients with chronic obstructive pulmonary disease (COPD) worsen health status [1,2] and are an important cause of consultations in primary care [3] and hospital admission [4]. Patients who have repeated exacerbations have an increased rate of decline in lung function [5,6]. and have a greater deterioration in health status over time [7]. The benefit of inhaled corticosteroids (ICS) in COPD has centred on their effect on exacerbations. ICS reduce both the frequency [8,9]. and severity [10] of exacerbations. In patients with mainly moderate to severe COPD, a meta-analysis showed that ICS caused an overall reduction of 30% in the rate of exacerbations [11].

International and UK guidelines recommend prescribing ICS for people with severe COPD with a post-bronchodilator FEV_1 _of less than 50% predicted and a history of frequent exacerbations [12,13]. The applicability of data from trials studying ICS in COPD to primary care populations is uncertain. Patients recruited to randomised clinical trials of ICS in COPD represent a small, selective and rather more severely affected fraction of patients with obstructive lung disease [14]. Eighty-five percent of patients with COPD in the UK and the Netherlands are managed exclusively in primary care, a figure increasing with the rising awareness and identification particularly of mild COPD in primary care [15,16]. Patients with COPD in primary care have often been prescribed ICS long-term, prior to the introduction of recent guidelines, and irrespective of their exacerbation frequency or lung function. It is not known whether withdrawing ICS in this population would lead to more exacerbations, and how withdrawal might affect daily symptoms and symptom profile at exacerbation.

In a randomised, double-blind, placebo-controlled study, we examined the effects of withdrawing ICS in people with COPD recruited in primary care. We tested the hypothesis that withdrawal of ICS in this population would lead to an increased number of COPD exacerbations, earlier onset of exacerbation, and a worsening of symptoms.

## Methods

### Practice and participant recruitment

We approached 36 general practices in east London and Essex to take part. Ethics approval for the study was obtained from the local regional ethics committee. We searched the medical record database at each practice to identify people aged 40 years and above, with a history of smoking, who had been prescribed ICS for a minimum of six months. We invited people who fulfilled these criteria to attend a recruitment interview. At interview we excluded people if they were on long term oral corticosteroids, not taking their prescribed ICS for at least four days a week, or had other chronic active lung disease or lung cancer. All patients provided written informed consent.

At recruitment we assessed lung function using the Vitalograph 2120, a calibrated fleisch-type pneumatachograph spirometer (Vitalograph Ltd, Buckingham, England). The spirometer was re-calibrated at each interview session. Patients were asked not to use their short-acting bronchodilator six hours before their appointment and not to use their long-acting bronchodilator from the night before. Bronchodilator reversibility was assessed 20 minutes after receiving five mg nebulised salbutamol. Patients with lung function consistent with international guidelines for the diagnosis of COPD were invited to join the study [13,16].: a post-bronchodilator FEV_1 _of less than 80% predicted, an FEV_1_/FVC ratio of less than 70% and a pre to post-bronchodilator change in FEV_1_of less than 15%. Patients with an FEV_1 _greater than 15% but a volume change of less than 200 mls were also included.

### Randomisation

Eligible patients who consented to participate at recruitment interview were given diary cards (see below) and instructed in their completion, followed by a two-week run in period before randomisation. At randomisation patients stopped their regular ICS and were allocated to fluticasone propionate 500 mcg, one puff twice daily using an accuhaler device, or identical placebo. Patients were allocated with minimisation to intervention and control using the programme MINIM v1.3. Minimisation factors were age, smoking status, pre-trial weekly dose of ICS, self-reported COPD exacerbation frequency and percentage predicted FEV_1_. Patients who had had an exacerbation treated by antibiotics or oral steroids in the preceding four weeks had their randomisation delayed by four weeks. Inhalers were given an alphanumerical code to conceal allocation. Study nurses and regular clinicians were blind to allocation throughout the study.

### Follow-up assessments

Follow-up assessments took place at three monthly intervals for a year at the patients' practice, and were conducted by trained study nurses. The nurse at follow-up verified symptom and health utilisation events recorded on diary cards with the patient. Spirometry was performed at each visit. Any adverse effects of ICS were recorded.

### Managing exacerbations

General practitioners were advised to manage exacerbations according to usual guidance with antibiotics and/or oral steroids. Decisions about stopping study inhalers and returning to usual (pre-randomisation) steroid inhalers were made by the general practitioner and patient. Patients who did return to their usual steroid inhaler after randomisation remained in the study, continued completing their diary cards, and were followed up for one year.

### Study outcomes

Our primary outcome was COPD exacerbation frequency. Secondary outcomes were time to first exacerbation (from diary cards and medical records), reported symptoms, peak expiratory flow rate and reliever inhaler use (from diary cards), and lung function and health related quality of life (at follow up visits).

We identified exacerbations by examining diary cards completed by patients, and their medical consultation and prescribing records.

Exacerbations were classified as a) unreported, b) moderate and c) severe.

a) An *unreported exacerbation *fulfilled symptom criteria on diary cards for a COPD exacerbation but was not managed with antibiotics or oral steroids.

b) A *moderate exacerbation *was defined as a COPD exacerbation treated with a course of antibiotics or oral steroids.

c) A *severe exacerbation *was defined as a COPD exacerbation treated with a course of antibiotics or oral steroids and resulting in hospital admission.

### Diary cards

All patients were given a daily diary card tested extensively in previous studies to record exacerbations for COPD [17]. Patients were taught how to complete the diary cards and were reminded at subsequent follow-up interviews. Respiratory symptoms were classified as 'major' (increasing breathlessness, sputum purulence and sputum production) or 'minor' (wheeze, cough, cold/nasal congestion, sore throat, fever). Using a previously validated classification [1,17], an exacerbation was defined as the presence for at least two consecutive days of increase in any two 'major' symptoms or increase in one 'major' and one 'minor' symptom according to criteria modified from Anthonisen and colleagues [18]. The first of the two consecutive days was taken as the day of onset of exacerbation. Patients were asked to report exacerbations to their usual clinician and not to the study team. Health care visits for exacerbation, daily reliever inhaler use, courses of oral steroids and antibiotics were recorded prospectively on the diary card. Patients were asked to record the highest of three post inhaler peak flow measurements at 10 am each day using a mini-Wright peak flow metre (Clement Clarke International, UK).

### Medical records

We examined the general practice medical records of all study patients for COPD consultations and prescribing data for COPD for the year following entry to the study. Written records were copied and computerised records printed out by blinded study nurses.

### Combining data from medical records and diary cards

A simple rule governed combination of data from the two sources: If the day of onset of an exacerbation in a diary card occurred within 4 weeks before or two weeks after the day of onset of an exacerbation recorded in medical records, this was classified as a single reported exacerbation, with the day of onset taken as that of the medical record.

### Quality of life

Patients completed the St George's respiratory questionnaire (SGRQ) at 0 and 12 months. The SGRQ has three weighted domains of symptoms, activities and impacts. SGRQ total score and sub scores range from 0 (no disability) to 100 (maximum disability) [19]. The EuroQol 5-D is a generic measure of health status comprising five dimensions and a visual analogue scale with 0 (maximum disability) and 100 (no disability) [20].

### Analysis

Sample size calculations were based on data from east London COPD patients reporting a median annual COPD exacerbation rate of 3 (range 1 to 8) [1]. We required 128 patients in each group to detect a 20% difference in the rate of exacerbation frequency between fluticasone and placebo groups with 80% power and significance of 5% over one year. The mean exacerbation frequency was analysed using a Poisson regression model adjusted for age, gender, season, smoking status, FEV_1 _severity, pre-trial dose of ICS and exacerbation frequency, and allowing for within-person correlation of events using generalised estimating equations.

Intervention and control group allocation were concealed during analyses. Analyses were carried out separately for total one-year duration of study (intention to treat) and time on randomised study inhaler (per protocol). We analysed our primary outcome in patients prescribed ICS in the non-mild group (predicted FEV_1 _percentage ≤ 50 or antibiotic/oral steroid courses in last year > 1 for COPD) with those who would not currently be prescribed ICS according to GOLD guidelines (predicted FEV_1 _percentage > 50 and antibiotic/oral steroid courses in last year ≤ 1). Time to first exacerbation was analysed using the unadjusted log rank test. Potential confounders were the same as for analyses of exacerbation frequency. Symptoms of shortness of breath, cough, wheeze and sputum production, reliever inhaler use and peak expiratory flow rate were analysed. We tested for differences between the fluticasone and placebo groups using generalised estimating equations with an autoregressive correlation structure to allow for within-patient correlation between outcomes on consecutive days. Symptoms were controlled for baseline level of symptom at randomisation (percentage of time with symptom between days -14 and -1 prior to randomisation of trial) and at first exacerbation (percentage of time with symptom between days -14 and -8 prior to onset of exacerbation) as well as smoking status, season and oral steroid and antibiotic use. Lung function and quality of life were analysed for each consecutive three-month period after randomisation using analysis of covariance adjusting for baseline levels of these outcomes.

## Results

### Practices and patients

Thirty-one of 36 (86%) general practices invited took part. Three practices declined, as they were involved in other trials. Two practices were excluded as information on ICS prescribing was poorly recorded.

**Figure 1:**
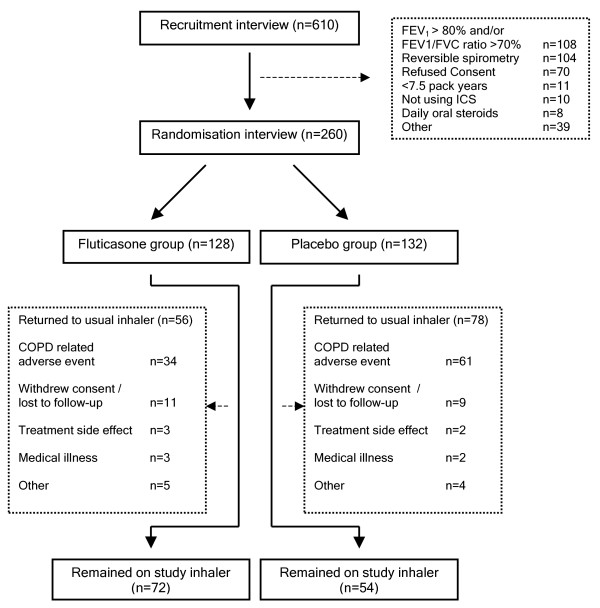
**Participant flow for the WISP trial**. ICS = inhaled corticosteroids.

Participant flow is shown in figure 1. Six hundred and ten people attended a recruitment interview, of which 212 (34.8%) did not fulfil spirometry inclusion criteria for COPD. Two hundred and sixty patients were randomised into the study, with 128 allocated to fluticasone 500 mcg twice daily and 132 to placebo. Characteristics of the two groups were well matched at baseline (table 1). The mean age was 67 years. Patients had smoked an average of 39 pack-years. Median duration of ICS use prior to randomisation was 8 years. Patients were prescribed a mean (SD) dose of 878 mcg (668 mcg) of beclomethasone equivalent ICS per day. The median (range, IQR) dose was 800 mcg (range 100 to 4000, 600). Higher doses of prescribed ICS (> 800 mcg per day) were significantly associated with a lower pre-trial percentage predicted FEV_1_, mean difference -8.8% (CI -13.3 to -4.2), more courses of antibiotics or steroids, mean difference 0.67 (CI 0.22 to 1.12) and the prescription of long acting beta 2 agonists, OR 4.0 (2.3 to 7.0).

**Table 1:** Baseline characteristics for fluticasone and placebo groups.

	Patients (n = 260)	
	Fluticasone n = 128	Placebo n = 132
Age (years) ^(a)^	67.6 (8.9)	67.3 (9.0)
Male n (%) ^(b)^	62 (48%)	74 (56%)
*Exacerbation history*		
Self-reported yearly COPD exacerbation rate	1.93 (1.52)	1.86 (1.57)
Antibiotics or steroids in last year^(c)^	1.59 (1.71)	1.48 (1.77)
*Smoking Status*		
Current smoker n (%)	52 (40.6%)	47 (35.6%)
Amount smoked (pack years)	40.0 (24.2)	38.8 (22.3)
*Medications*		
Duration of inhaled medications (years)	8.7 (7.0)	8.2 (5.6)
Daily Prescribed Dosage of ICS^(d) ^(mcg)	947 (784)	812 (531)
On LABA medication n (%)	45 (35.1%)	42 (31.8%)
Influenza vaccine in winter	104 (81.2%)	106 (80.3%)
*Lung Function*		
Pre bronchodilator FEV_1 _(litres)	1.22 (0.54)	1.32 (0.54)
Post bronchodilator FEV_1 _(litres)	1.31 (0.55)	1.40 (0.56)
Post bronchodilator FEV_1 _(% predicted)	53.2 (18.2)	55.0 (17.1)
Reversibility (% change in FEV_1_)	8.1 (8.0)	7.2 (6.8)
Post bronchodilator FVC (litres)	2.40 (0.77)	2.56 (0.88)
Peak expiratory flow rate (L/min)	220 (90)	235 (90)
*Health Related Quality of Life*		
SGRQ Total (%)	52.6 (20.2)	47.2 (19.4)
EuroQol 5D total score	0.64 (0.29)	0.68 (0.30)
EuroQol 5D visual analogue scale	60.6 (18.8)	61.1 (20.8)
*MRC Dyspnoea Scale*		
Scale 3 or greater (%)	77 (60%)	66 (50%)
*Chronic Symptom Profile*^(d)^		
Shortness of Breath n (%)	96 (75%)	95 (72%)
Sputum Production n (%)	69 (54%)	78 (59%)
Wheeze n (%)	60 (47%)	59 (45%)
Cough n (%)	72 (56%)	69 (53%)

(a) Continuous data is displayed as mean value and standard deviation.

(b) Categorical data is displayed as number of cases (n) and percentage of group for fluticasone or placebo.

(c) Patient prescriptions at general practice/hospital/accident and emergency/outpatient visits for antibiotics and/or steroids for COPD exacerbation.

(d) Dosage of daily inhaled corticosteroid prescribed is stated a s beclomethasone equivalent in micrograms.

(e) Chronic symptom profile at recruitment interview. LABA = Long acting beta 2 agonist.

47,460 days of diary card records (days with data) were returned by patients after randomisation, equivalent to a completion rate of 88.2% of total study days. The proportion of completed diary cards was similar in both groups: fluticasone 87.7%, placebo 88.8%. The completion of diary card days in patients returning to their usual steroid inhaler was 63.2% in the fluticasone group and 57.3% in the placebo group.

### Primary outcome

#### Exacerbation frequency

##### a) Exacerbations for duration of study (intention to treat analysis)

When data for all 260 patients for the one year duration of the study were included in the analysis, irrespective of whether patients had returned to their usual ICS inhaler, the mean exacerbation frequency was 2.92 (CI 2.47 to 3.37) for the fluticasone group and 3.13 (CI 2.61 to 3.65) for placebo, adjusted RR 1.11 (0.91 to 1.36), P = 0.298. The mean exacerbation frequency for reported (moderate and severe) exacerbations was 1.91 (CI 1.55 to 2.28) for fluticasone and 2.24 (CI 1.82 to 2.66) for placebo, adjusted RR 1.25 (0.95 to 1.58), P = 0.067.

##### b) Exacerbations on randomised treatment (per protocol analysis)

The mean duration of exposure to randomised treatment was 235 days for fluticasone group and 179 days for placebo group (table 2). Whilst taking randomised treatment, patients in the fluticasone group had 279 exacerbations (59.9% reported); those in the placebo group had 293 exacerbations (66.2% reported). Whilst taking randomised treatment patients were significantly more likely RR 1.48 (CI 1.17 to 1.86, P < 0.001) to suffer an exacerbation whilst taking placebo than whilst taking fluticasone. For reported (moderate and severe) exacerbations, the relative risk of exacerbating on placebo inhaler was greater: RR 1.63 (CI 1.23 to 2.17), P < 0.001.

**Table 2:** Relative risk of COPD exacerbations stratified by severity in patients randomised to fluticasone and placebo groups over one year.

	**COPD exacerbations**					**Relative risk of COPD exacerbation for patients randomised to placebo or fluticasone (n = 260)**				
	**Fluticasone (n = 128)**		**Placebo (n = 132)**		**Mean days of patient exposure**	**Exacerbation grouping**	**Incidence rate ratio**	**95% confidence interval**		**adjusted p-value**
**Exacerbations while in trial**	Unreported	129	Unreported	116	235 in fluticasone, 179 in placebo	All	**1.11**	0.91	1.36	0.298
	Moderate	224	Moderate	276						
	Severe	21	Severe	22		Moderate and severe only	**1.25**	0.96	1.58	0.067
	**Total**	**373**	**Total**	**413**						

**Exacerbations while on randomised treatment**	Unreported	112	Unreported	99	371 in both groups	All	**1.48**	1.17	1.86	0.001
	Moderate	158	Moderate	182						
	Severe	9	Severe	12		Moderate and severe only	**1.63**	1.23	2.17	0.001
	**Total**	**279**	**Total**	**293**						

One-hundred and two (39.2%) of patients did not meet GOLD guidelines for prescription of ICS in COPD (predicted FEV1 > 50% and courses of antibiotics/oral steroids ≤ 1 per year for COPD). Before randomisation, patients in this mild group had less severe airflow obstruction: FEV_1_/FVC ratio mean difference 14.9% (CI 11.6 to 18.3), were prescribed a lower dose of ICS: mean difference 337 mcg (CI 186 to 489) and reported less shortness of breath OR 0.13 (CI 0.01 to 0.22) than in the more severe group (predicted FEV_1 _percentage ≤ 50 and/or courses of antibiotics/oral steroids > 1 per year for COPD). The risk of exacerbating after withdrawal of ICS whilst randomised to treatment in patients with mild COPD disease was significantly increased: RR 1.94 (CI 1.20 to 3.14). For the remaining 158 (60.8%) of COPD patients, the risk of exacerbation after withdrawal was non-significantly raised (RR 1.24, CI 0.96 to 1.41). However, the exacerbation risk in this group was not significantly different to the exacerbation risk in the group not fulfilling GOLD criteria for ICS prescription (P-value between groups = 0.1).

### Secondary outcomes

#### Time to first exacerbation

**Figure 2:**
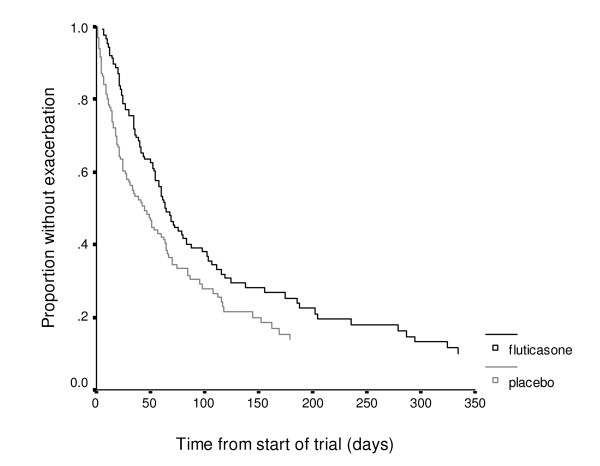
Kaplan-Meier curve for time to first COPD exacerbation.

190 (73.1%) of the 260 patients had at least one exacerbation whilst randomised to the study inhaler. The median time to first exacerbation was 19 days shorter for patients randomised to placebo: 63 days (CI 53 to 74) for the fluticasone group, and 44 days (CI 29 to 59) for the placebo group, (Log Rank 3.81, P = 0.05). Early exacerbation was more common in the placebo group with 56 (42%) of patients in the placebo group exacerbating in the first month after randomisation compared to only 30 (23%) in the fluticasone group. A Cox regression analysis adjusted for variables controlled for in exacerbation frequency analyses showed an increased hazard ratio for first exacerbation in the placebo group, OR 1.43 (CI 1.08 to 1.96) compared to fluticasone. Cumulative survival for time to first exacerbation is shown in figure 2. In the sub-group of 102 patients in our study that did not fulfil GOLD guidelines criteria for ICS, their remains an increased hazard ratio for first exacerbation in the placebo group OR 1.68 (CI 0.96 to 2.96). No significant differences was found between hazard ratios in those that did and did not fulfil GOLD guidelines criteria for ICS

#### Symptoms, peak expiratory flow rate and reliever inhaler use

##### a) For duration of study

**Figure 3:**
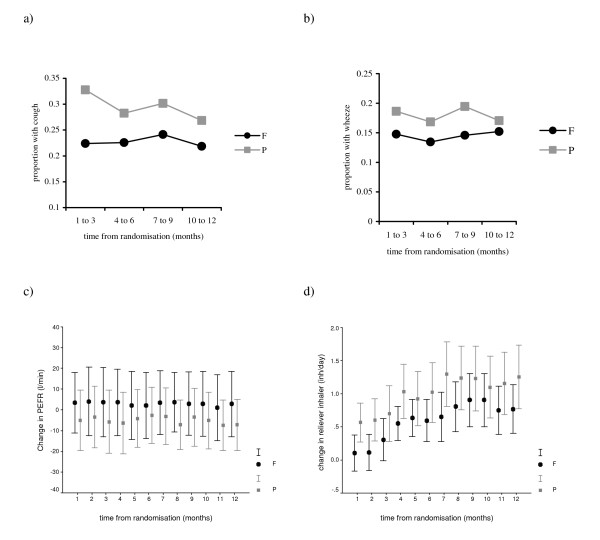
**Symptoms, change in peak expiratory flow rate (PEFR) and change in reliever inhaler use in fluticasone (F) and placebo (P) during study**. Figures a) and b) show the proportion of patients in each drug group recording cough and wheeze on their diary card. Reporting of cough was significant between groups in first 3 months only: OR 1.95 (CI 1.16 to 3.29) P < 0.05. Wheeze was significant between groups for whole year: OR 1.83 (CI 1.06 to 3.18) P < 0.05. Figures c) and d) show mean change from baseline for PEFR and reliever inhaler use. Baseline for PEFR and reliever inhaler use was calculated from the mean value from day -8 to -1 prior to randomisation. PEFR was not significant between groups at 12 months: Mean difference 7.86 l/min (CI -1.45 to 17.17) P > 0.05. Reliever inhaler was significant in first month only: Mean difference 0.53 inh/day (CI 0.06 to 1.00) P < 0.05. (inh/day = inhalations of reliever inhaler per day).

Patients recorded a significant increase in wheeze whilst randomised to placebo for the whole year, OR 1.83, (CI 1.06 to 3.18). Reporting of shortness of breath, OR 2.11, (CI 1.25 to 3.57) and cough OR 1.95 (CI 1.16 to 3.29) was significantly greater in the placebo group for the first 3 months of the study only (figure 3). Reporting of sputum production and other symptoms were similar in both groups. Patients in the placebo group used their reliever inhaler more frequently, mean difference, 0.45 inhalations/day, (CI -0.21 to 1.11). This was significant in the first month after randomisation; mean difference 0.53 inhalations/day (CI 0.06 to 1.00). There was a persistent fall in mean peak expiratory flow rate in the placebo group over 12 months, mean difference, -7.86 litres/min, (CI -17.17 to 1.45).

##### b) For first exacerbation after randomisation

**Figure 4:**
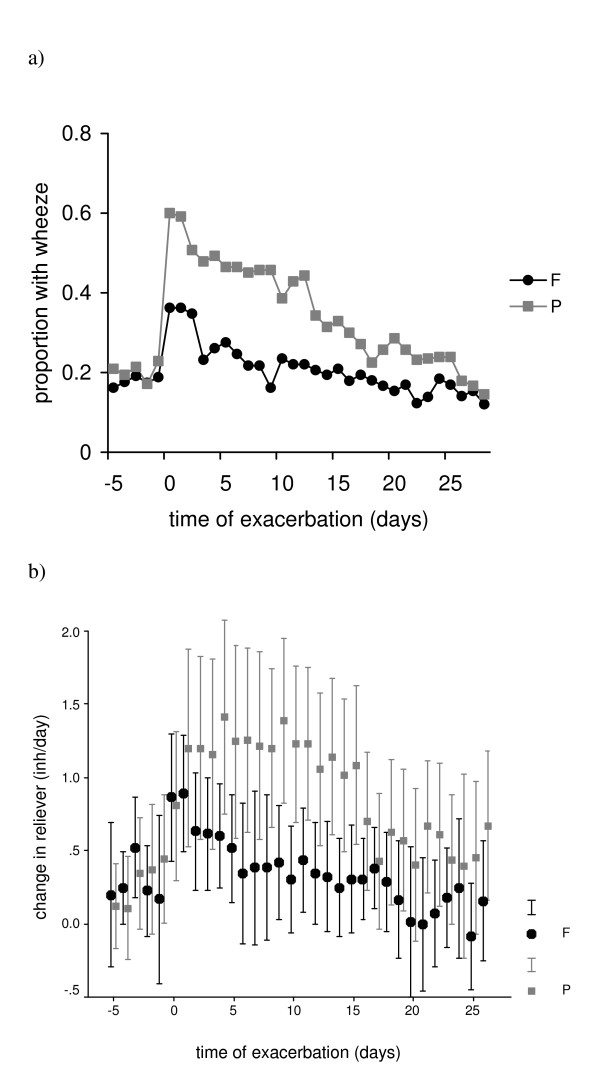
**Wheeze and reliever inhaler use in fluticasone (F) and placebo (P) groups at time of first COPD exacerbation (-8 to 28 days)**. Day 0 is the first day of exacerbation as defined in our methods section. Figure a) shows the proportion of patients with wheeze on each day for fluticasone and placebo groups. Reporting of wheeze was significant 28 days from onset of exacerbation: OR 1.85, (CI 1.35 to 2.53) P < 0.05. Figure b) show mean change from baseline for daily reliever inhaler use in each group. Baseline reliever inhaler use was calculated from the mean value from day -14 to -8 prior to onset of exacerbation. Difference in reliever inhaler use between groups was significant day -7 to 28: Mean difference 0.44 inh/day (CI 0.33 to 0.55) P < 0.01. (inh/day = inhalations of reliever medication per day).

We analysed diary records for patients' first exacerbation after randomisation. Reporting of shortness of breath, OR 1.64, (CI 1.22 to 2.22) and wheeze, OR 1.85, (CI 1.35 to 2.53), was significantly greater in the placebo group during exacerbation. Sixty-two percent of patients in the placebo group reported wheeze on the first day of exacerbation compared to 36% in the fluticasone group (figure 4).

#### Lung Function and Quality of Life

**Figure 5:**
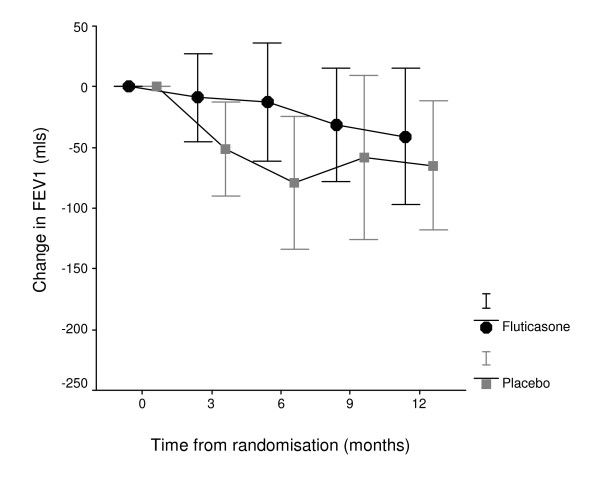
**Change in forced expiratory volume in one second (FEV_1_) at three monthly assessments in study**. Baseline FEV_1 _was value at randomisation. Mean difference for FEV_1 _(mls) at 12 months: fluticasone -41mls, placebo -64mls (P = 0.44).

A decrease in FEV_1 _was found in both groups after withdrawal (figure 5). The decrease in FEV_1 _from baseline was greater in placebo than fluticasone for all four assessments after randomisation. No significant differences in FEV_1_, FVC or FEV_1_/FVC ratio was found between placebo and fluticasone at.12 months. Mean difference for FEV_1 _(mls) at 12 months: fluticasone -41 mls, placebo -64 mls (effect size: % partial eta^2 ^squared 0.7%, sig. P = 0.44).

SGRQ total scores showed a small but non-significant decrease (i.e. better health status) in both groups after one year. There were no significant differences found at 6 or 12 months for SGRQ total score and sub-scores between fluticasone and placebo. EuroQol 5-D total and visual analogue scale showed no significant difference at 12 months for fluticasone and placebo. All scores were adjusted for baseline score, smoking status and season (table 3).

**Table 3:** Quality of life scores in patients randomised to fluticasone or placebo.

	**Fluticasone**	**Placebo**		
	Mean difference at 12 months	Mean difference at 12 months	Effect size % partial eta2	Significance between groups
**SGRQ total score**	-0.98	-1.43	< 0.1%	P = 0.83
**EQ5D total score**	-0.03	0.03	0.2%	P = 0.66
**EQ5D VAS**	-2.16	-0.29	< 0.1%	P = 0.97

(a) SGRQ = St George's respiratory questionnaire.

(b) EQ5D = EuroQol 5D generic questionnaire.

(c) Significance between groups was assessed using analysis of co-variance.

#### Return to usual steroid inhaler

Of the 260 patients in the study, 126 (48.5%) completed the study on the randomised inhaler, with 134 returning to their usual ICS inhaler. Sixty-one (46%) patients in the placebo group returned to their usual inhaler as a direct result of an exacerbation or self-reported respiratory symptom deterioration compared to 34 (26%) in the fluticasone group (OR 2.35, CI 1.38 to 4.05). Patients with a lower percentage predicted FEV_1 _were more likely to return to their usual inhaler, mean difference 5.08%, (CI 0.73 to 9.44). Patients were exposed to randomised treatment for 179 days (CI 153 to 206) in the placebo group and 235 days (CI 209 to 262) in the fluticasone group.

#### Adverse events and adverse effects of treatment

There were three COPD related deaths, all occurring in the fluticasone group. These patients all had severe COPD on spirometric criteria and had frequent exacerbations requiring recurrent hospital admissions prior to entry into trial. There was no significant difference in reporting of skin bruising, thinning of skin, sore throat, oral thrush or hoarseness of voice between fluticasone and placebo groups during the study.

## Discussion

### Summary

We report the first randomised placebo-controlled study of withdrawal of long-term inhaled corticosteroids in patients with COPD recruited in primary care. Risk of COPD exacerbation was increased in patients withdrawn from ICS, significantly so in the per protocol analysis of patients on randomised treatment. Withdrawal also led to earlier exacerbation, with a rapid deterioration in symptoms. In the year following withdrawal, patients reported more wheeze and a significant increase in the use of their reliever inhaler in the first month. Patients were more likely to return to their usual ICS following an exacerbation in the withdrawal group. Patients with mild COPD, for whom current guidelines do not recommend ICS, also had an increased risk of exacerbation after withdrawal.

### Strengths and limitations of this study

Our study had a number of strengths. First, we systematically screened general practice patients prescribed long-term inhaled steroids to identify a representative sample of primary care COPD patients with a wide range of exacerbation frequencies (0 to 10 per year) and severity (percentage predicted FEV_1 _13 to 80%). The majority of our patients were managed entirely in primary care, with only 13% seen in a respiratory clinic and 14% admitted to hospital with a COPD exacerbation in the year before the study, exactly matching national estimates [15,16]. Second, our spirometric and smoking history recruitment criteria were consistent with all other major trials of steroid withdrawal in COPD [21,22] and ensured our sample was unlikely to be contaminated with patients with asthma, which might lead to overestimation of the effect of ICS withdrawal. Thus, the demographics of our COPD population with respect to age and pack years of smoking were almost identical to other major trials of steroid withdrawal, although our study mean FEV_1 _percent predicted (55%) was marginally higher, as might be expected in COPD patients recruited in primary care. Third, using diary cards with a high completion rate, and examining medical records, allowed us to capture a near complete picture of all exacerbations, including those unreported to health care. Up to 50% of COPD exacerbations pass unreported to a clinician [17], and perhaps more in a primary care population. Reliance solely on patient recall of COPD exacerbations is common but risks underestimation [8,23]. As a consequence, the exacerbation rate in our study was greater than in some previous intervention studies [18]. Fourth, we chose a pragmatic study design, which enabled us to obtain an accurate and generalisable assessment of the effects of withdrawing ICS in primary care COPD patients. Fifth, examining a range of outcomes (exacerbations, symptoms, reliever inhaler use, reversion to original ICS inhaler) meant we were able to report a consistent effect across outcomes, adding credibility to our results.

Allowing the patients' regular clinician to manage COPD exacerbation without intervention from the study team had both advantages and disadvantages. This approach gave a useful indication of the patients' preferences for their original inhaler should ICS be withdrawn in routine practice outside the setting of a trial and showed a differential cessation rate of study medication (26% fluticasone group, 46% placebo group) consistent with favourable effect of ICS on other outcomes in our study. However, differential cessation rates made it important to reduce potential bias in the analysis by detailed follow up of all patients for the duration of the study, and by reporting separate analyses for exacerbations occurring whilst patients were taking study medication (per protocol) and for the duration of the study (intention to treat).

### Comparison with other studies

All previous randomised controlled studies on this topic have reported the effects of withdrawing ICS in patients with COPD in secondary care [21,22,24,25]. Our findings in primary care patients are broadly similar. The COPE study examined time to first exacerbation in 244 COPD patients from a single hospital outpatient clinic with a shorter 6-month follow-up [21]. This study reported a hazard ratio of a first exacerbation of 1.5 between placebo and ICS groups, similar to our finding for patients on randomised treatment. The COSMIC randomised 373 COPD patients taking salmeterol/fluticasone combination to withdrawal of fluticasone over one year [22]. Their population comprised a more severe group of patients with two or more exacerbations per year prior to randomisation. In contrast to our findings, the COSMIC study reported a significant increase in mild, but not moderate and severe exacerbations in those who withdrew from fluticasone, a difference that may reflect differences in definitions and data collection methods. O'Brien et al conducted a short 12 week crossover study in 24 men with severe irreversible COPD and found deterioration in lung function and increase in exercise-induced dyspnoea [24]. Jarad et al also found high exacerbation rates in an observational study as part of the run-in for the ISOLDE study, reporting exacerbation in 38% of patients who had stopped their prescribed ICS before randomisation compared to only 6% in the chronically untreated group over a seven-week period [25]. One non-randomised, unblinded study set in primary care found an increase in patients having an exacerbation of COPD after ICS withdrawal [26].

Our study did not detect any significant difference in quality of life after withdrawing inhaled corticosteroids. This was unexpected given the effect on exacerbations in our study and the known relationship between COPD exacerbations and quality of life [1]. Our findings may reflect the fact that a number of our patients returned to their usual inhaler before the end of the study which could have ameliorated the effects of the study drug. Previous studies have found differing results on quality of life. The COPE study did show a deterioration in the total SGRQ score after six months between placebo and inhaled corticosteroid group. However, the COSMIC study only showed a deterioration of SGRQ total score in both arms of the study but no significant difference between groups after one year.

### Clinical implications

Withdrawal of ICS caused a sustained significant increase in wheeze throughout the year. Cough and shortness of breath was significantly increased for the first three months. Patients describing these changes did so early after randomisation, by day three for shortness of breath, and day four for cough and wheeze. These findings were similar to the COSMIC study that also reported an acute deterioration in symptoms. However, we only found a difference for shortness of breath for the first three months of the study and found no significant difference in the reporting of sputum production. Wheeze was not recorded in their study. There was a notable effect on lung function on withdrawal. An immediate and sustained drop in peak expiratory flow rate throughout the study was noticed from day two of study. It is likely that the effects on symptoms, reliever inhaler use and peak expiratory flow rate on withdrawal may have been significant but for patients returning to their usual inhaler during the study.

Our findings of a consistent range of adverse effects of ICS withdrawal from COPD patients drawn from primary care provide useful confirmation of, but also extend analyses from secondary care populations [21,22,24,25]. These data support and strengthen the recommendations on use of ICS in patients with moderate and severe disease. Many of our patients did not fulfil GOLD criteria for prescription of ICS, but had been prescribed them nonetheless by their general practitioners. Considerable uncertainty surrounds the pharmacological management of such patients. 151 (58%) patients had a predicted FEV_1 _> 50%, and 158 (60%) had less than two COPD exacerbations per year. 102 (39%) of patients had both a predicted FEV_1 _> 50% and exacerbation rate of less than two a year. Withdrawing ICS in these 102 patients exposed them to an increased risk of exacerbation comparable with patients with moderate and severe COPD. Why patients with mild COPD faired no better is unclear. All patients lacked reversibility of FEV_1 _after high-dose nebulised salbutamol at study entry. We examined percentage change in FEV_1 _before and after bronchodilator challenge as a confounder to risk of exacerbation and time to first exacerbation and found that greater bronchodilator reversibility did not predict earlier exacerbation. Milder COPD patients in our subgroup had reported significantly less pre-trial shortness of breath and had less airflow obstruction. The milder COPD patients are therefore likely to represent a subgroup with less chronic airways inflammation than in the severe group [27]. Despite this, patients in the mild group reported high levels of shortness of breath and wheeze similar to that of the severe group at first exacerbation. Our patients had been taking ICS for many years, it is possible that these patients may have become 'unmasked' from the possible protective effects of ICS on withdrawal. This phenomenon would not be seen in prospective studies of ICS or in studies on withdrawal of ICS with a short run-in period [21,22].

Secondly, it is noted that the milder group had been prescribed significantly lower doses of pre-trial ICS. There has been no dose-response relationship established for ICS in patients with COPD. In our study, patients in the severe group may have had a greater step-down of ICS when randomised to placebo. Conversely patients in the mild group may have had an increase in their ICS dose when randomised to fluticasone. The dose of pre-trial ICS does have an effect on our primary outcome. Patients with ICS dose higher than 800 mcg had 1.52 (95% CI 1.19 to 1.95) times more exacerbations than those on a lower dose of ICS (< 800 mcg). However, the effect of the study intervention when adjusted for pre-trial ICS remains virtually unchanged. When we repeat the analysis but with adjustment for all the covariates including FEV_1 _severity and pre-trial exacerbation frequency, ICS dose no longer significantly affects outcome, the relative risk has fallen to 1.1 (95% CI 0.78 to 1.56). The likely interpretation is that pre-trial ICS dose is a marker of severity which affects exacerbation rate, but is linked to all the other markers of severity as well. Our finding that the risk of exacerbation was also raised in patients with mild COPD after ICS withdrawal raises the question of whether these patients should be recommended these drugs. Our findings come from a sub-group analysis in a withdrawal study and therefore may be treated with caution, but are strengthened by using diary cards and examination of patient medical records.

## Conclusion

In summary, withdrawal of long term ICS in patients with COPD from primary care increases the risk of exacerbation, shortens the time to first exacerbation and leads to worsening wheeze. The reversion of larger numbers of patients on placebo to pre-randomisation ICS following exacerbations ameliorated this risk over the one year period of the trial. Careful consideration of the risks and benefits of withdrawal of ICS should be made. Patients with COPD stopping long-term ICS should be monitored closely for deterioration, irrespective of their baseline FEV_1 _or exacerbation frequency.

## Competing interests

Author 1: Aklak B. Choudhury. No conflicts of interest declared

Author 2: Carolyn M. Dawson. No conflicts of interest declared

Author 3: Hazel E. Kilvington. No conflicts of interest declared

Author 4: Sandra Eldridge. No conflicts of interest declared

Author 5: Wai-Yee James. No conflicts of interest declared

Author 6: Jadwiga A. Wedzicha Yes.

JAW has received honoraria for lectures at meetings and/or attendance at advisory boards from GSK, Boehringer Ingelheim, Astra Zeneca, Bayer, Roche, Vitalaire, Aventis Pasteur and Aventis Pharma and she has received research grants in the last 3 years totalling $450,000 from Glaxo Smith Kline, $550,000 from Boehringer Ingelheim, £25,000 from Astra Zeneca and $300,000 from Aventis Pateur.

Author 7: Gene S. Feder. No conflicts of interest declared

Author 8: Chris J. Griffiths Yes

CG received £80,000 from Pfizer for a study of the relationship between ethnicity and response to antihypertensive medication (Anglo-Scandinavian Cardiac Outcomes Trial. ethnicity sub-study).

## Authors' contributions

AC, JW, CG and GF conceived and designed study. AC, CD, HK and WJ conducted participant interviews, collected exacerbation data from practices, analysed symptoms on diary cards. SE performed statistical analysis. AC and CG helped coordinate the study. AC, JW, CG and GF helped to draft the final manuscript. All authors read and approved the final manuscript.
